# The Human Autoantibody Response to Apolipoprotein A-I Is Focused on the C-Terminal Helix: A New Rationale for Diagnosis and Treatment of Cardiovascular Disease?

**DOI:** 10.1371/journal.pone.0132780

**Published:** 2015-07-15

**Authors:** Sabrina Pagano, Hubert Gaertner, Fabrice Cerini, Tiphaine Mannic, Nathalie Satta, Priscila Camillo Teixeira, Paul Cutler, François Mach, Nicolas Vuilleumier, Oliver Hartley

**Affiliations:** 1 Department of Human Protein Sciences, Faculty of Medicine, University of Geneva, Geneva, Switzerland; 2 Division of Laboratory Medicine, Department of Genetics and Laboratory Medicine, Geneva University Hospitals, Geneva, Switzerland; 3 Department of Pathology and Immunology, Faculty of Medicine, University of Geneva, Geneva, Switzerland; 4 Pharmaceutical Sciences, Pharma Research and Early Development, F.Hoffmann-La Roche, Basel, Switzerland; 5 Division of Cardiology, Foundation for Medical Researches, University of Geneva, Geneva, Switzerland; University of Bologna, ITALY

## Abstract

**Background:**

Cardiovascular disease (CVD) is the leading cause of death worldwide and new approaches for both diagnosis and treatment are required. Autoantibodies directed against apolipoprotein A-I (ApoA-I) represent promising biomarkers for use in risk stratification of CVD and may also play a direct role in pathogenesis.

**Methodology:**

To characterize the anti-ApoA-I autoantibody response, we measured the immunoreactivity to engineered peptides corresponding to the different alpha-helical regions of ApoA-I, using plasma from acute chest pain cohort patients known to be positive for anti-ApoA-I autoantibodies.

**Principal Findings:**

Our results indicate that the anti-ApoA-I autoantibody response is strongly biased towards the C-terminal alpha-helix of the protein, with an optimized mimetic peptide corresponding to this part of the protein recapitulating the diagnostic accuracy for an acute ischemic coronary etiology (non-ST segment elevation myocardial infarction and unstable angina) obtainable using intact endogenous ApoA-I in immunoassay. Furthermore, the optimized mimetic peptide strongly inhibits the pathology-associated capacity of anti-ApoA-I antibodies to elicit proinflammatory cytokine release from cultured human macrophages.

**Conclusions:**

In addition to providing a rationale for the development of new approaches for the diagnosis and therapy of CVD, our observations may contribute to the elucidation of how anti-ApoA-I autoantibodies are elicited in individuals without autoimmune disease.

## Introduction

Despite increasing public awareness and major therapeutic progress, cardiovascular disease (CVD) remains the leading cause of morbidity and mortality worldwide [1]. Calls have been made to develop improved strategies for prevention, especially risk stratification [1, 2] and treatment [3] of both CVD and atherosclerosis, its underlying cause. Autoantibodies represent potentially useful biomarkers in risk stratification for atherosclerosis and CVD, some of them providing strong prognostic information independently of established risk factors [4].

Apolipoprotein A-I (ApoA-I), the major protein constituent of high density lipoprotein (HDL), is a 28 kDa protein whose lipid-free structure consists of six alpha-helices arranged in two bundles, an N-terminal four-helix bundle and a C-terminal two-helix bundle [5, 6]. Although the respective contributions of the lipid versus the lipoprotein fraction towards the anti-atherogenic effects of HDL is still debated, several studies indicate that lipid-free ApoA-I itself can perform many of the atheroprotective activities ascribed to HDL, including reverse cholesterol efflux and inhibition of different pro-inflammatory, pro-oxidant and pro-thrombotic pathways [7, 8].

The link between anti-ApoA-I autoantibodies of immunoglobulin G (IgG) class and CVD was first noted in studies of patients with autoimmune diseases [9–13] and initially linked to a loss of atheroprotective HDL functions [9–11]. Subsequently, anti-ApoA-I IgG was shown (i) to be an independent predictor of poor cardiovascular outcome in several different populations at risk for CVD without concomitant autoimmune disease [14–17], and (ii) to provide incremental prognostic information over traditional risk factors for CVD [14–16, 18]. While the mechanism by which anti-ApoA-I autoantibodies are elicited is not currently understood, a series of cellular and animal studies have highlighted a causal role for anti-ApoA-IgG in atherogenesis, suggesting that it might represent a target for therapeutic intervention. Passive immunization of apoE^-/-^ mice with anti-ApoA-I IgG was shown to increase both atherosclerotic lesion size as well as histological features of atherosclerotic plaque vulnerability [15]. Several different potential pathogenic mechanisms have been proposed [12, 15, 17, 19–21], including (i) induction of proinflammatory cytokine release from macrophages [12, 15, 19] through interaction with the TLR2/CD14 complex [19], (ii) a pro-arrhythmogenic effect on cardiomyocytes *in vitro* [17, 20], and (iii) the induction of dysfunctional HDLs *in vivo* [21].

In this study we set out to characterize the anti-ApoA-I autoantibody response using a series of synthetic peptides derived from the different helical regions of the protein, with the aim of identifying candidate mimetic peptides suitable for use in diagnosis and/or therapy of atherosclerosis and CVD.

## Materials and Methods

### Ethics Statement

The research Ethics Committee of Geneva University Hospitals approved the study protocol. All patients gave written informed consent before enrolment.

### Clinical Study Design

The clinical study presented here is ancillary to work derived from a previously published prospective single center study exploring the diagnostic accuracy of anti-ApoA-I IgG for type I NSTEMI diagnosis on 138 patients presenting to the emergency room for acute chest pain and meeting the required power of 90% [14]. As patients’ plasma was no longer available for six patients, only 132 patients were available for analyses. To minimize the power impact of this sample shortage, we used a composite endpoint consisting of acute ischemic coronary etiology defined in the presence of type 1 NSTEMI (n = 22) or unstable angina (n = 7), according to the universal criteria of acute myocardial infarction AMI [14, 22]. The study endpoint was established by two independent senior cardiologists who were blinded to biochemical results. If patients did not fulfill the universal criteria of AMI in the presence of cTnI elevation, a non-ischemic etiology was concluded only after exclusion of ischemia using myocardial scintigraphy or cardiac magnetic resonance imaging or after exclusion of a significant culprit coronary lesion by coronary angiography.

Inclusion criteria consisted of chest pain lasting more than 5 min, regardless of age and gender, without ST-segment elevation on ECG defined by the absence of ST⁄T abnormalities or dynamic changes, such as non-persistent ST-segment elevation, ST depression, T-wave abnormalities or no ECG changes. Exclusion criteria consisted of STEMI, chest pain for a duration of less than 5 min, prior hospitalization within 48 hours, known autoimmune diseases such as rheumatoid arthritis (RA), systemic lupus erythematosous (SLE) or anti-phospholipid syndrome (APS), known HIV or clinically patent signs of heart failure.

### Antibodies and patients plasma

Goat polyclonal anti-human ApoA-1 IgG was obtained from Academy Bio-Medical Company. Patient plasma samples used in this study were archived from a previously published prospective single-centre study of 138 clinically well-characterized patients presenting at the emergency room for acute chest pain (ACP) [14], as described above. Blood was taken upon patient admission, centrifuged, aliquoted, and stored at -80°C until analysis.

### Determination of human antibodies to ApoA-I and ApoA-I derived peptides by ELISA

Anti-ApoA-I IgG autoantibodies in plasma were measured as described previously [12–18]. Briefly, Maxisorp plates (Nunc) were coated with purified and delipidated human ApoA-I, diluted in carbonate buffer pH 9.7 (20 μg/ml; 50 μl/well), for 1 h at 37°C. The same procedure was used for the engineered peptides. After three washes with PBS/ 2% (w/v) BSA (100 μl/well), all wells were blocked for 1 h with PBS/ 2% (w/v) BSA at 37°C. Samples were diluted 1:50 in PBS/ 2% (w/v) BSA and incubated for 60 min at 37°C. Samples at the same dilution were also added to a non-coated well to assess individual non-specific binding. After six further washes, 50 μl/well of alkaline-phosphatase conjugated anti-(human IgG) (Sigma-Aldrich), diluted 1:1000 in PBS/ 2% (w/v) BSA, was incubated for 1 h at 37°C. After six more washes (150 μl/well) with PBS/ 2% (w/v) BSA, the phosphatase substrate *p*-nitrophenyl phosphate (50μl/well; 1mg/ml; Sigma—Aldrich) dissolved in 4.8% (w/v) diethanolamine (pH 9.8) was added. Each sample was tested in duplicate and *A* 405nm was determined after 20 min of incubation at 37°C (VERSAMax; MolecularDevices). The corresponding non-specific binding was subtracted from the mean absorbance for each sample. As in previous studies [12–18], positive immunoreactivity of human samples to ApoA-I was prospectively defined by an index >37%. This value was also used to define positive immunoreactivity to mimetic peptides, as defined upon receiver operating characteristic (ROC) curve analyses, and shown to provide an identical prevalence of positive immunoreactivity levels 11% (15/132) to native ApoA-1 and 11% (14/132) to F3L1 peptide (data not shown). Repeatability and reproducibility were determined at two levels. At a high level (*A* 405nm = 1.2, i.e. twice the cut-off value), the intra- and inter-assay coefficients of variation were 10% (*n* = 10) and 17% (*n* = 10) respectively. At the cut-off level, the intra- and inter-assay coefficients of variation were 16% (*n* = 10) and 12% (*n* = 8) respectively.

### Peptide synthesis

Peptide fragments were synthesized according to a standard Fmoc-protocol on Rink amide AM resin (Novabiochem) using a Prelude synthesizer (Protein Technologies). For peptide F3L1, a pair of orthogonal protecting groups (allyl / allyloxycarbonyl) was used for the glutamic acid and lysine residues utilized to form the lactam bridge. At the end of resin elongation, these protecting groups were removed according to the procedure of Kates *et al*. [23]. On-resin lactam bridge formation [24], monitored by Kaiser ninhydrin test, was carried out with 3 equivalents of 6-chloro-benzotriazole-1-yl-oxy-tris-pyrrolidino-phosphonium hexafluorophosphate and 9 equivalents of N,N-diisopropylethylamine over 48 h at 37°C. Peptides were cleaved from the resin with 90% trifluoroacetic acid, 5% phenol, 2.5% water and 2.5% triisopropylsilane, precipitated in diethylether, purified (to >95%) by reverse-phase HPLC, and lyophilized. The masses for each peptide were verified by mass spectrometry.

### CD spectroscopy

Experiments were carried out in 0.1 cm quartz cell using a Jasco J-710 spectrometer with 100 μM peptide solutions (1.25% trifluoroethanol in water) at 20°C.

### Pro-inflammatory cytokine release

Human monocytes were isolated from buffy coats from healthy donors at the Geneva University Hospitals Blood Transfusion Center and differentiated into macrophages by 24 h incubation with IFN-γ (500 U/ml) in complete RPMI-1640 culture medium (10% heat-inactivated FCS, 50 μg/ml streptomycin, 50 U/ml penicillin, 2 mM L-glutamine), as previously described [19]. Assays of anti-ApoA-I IgG-mediated release of IL-6 and TNF-α from human monocyte-derived macrophages was carried out as described previously [12, 15, 19] using a previously determined optimal concentration of polyclonal goat anti-ApoA-I IgG (40 μg/ml). Endotoxin contamination in the assay was excluded using the limulus amebocyte lysate Endochrome assay [19]. Where indicated, anti-ApoA-I IgG was pre-incubated with peptide F3L1 (1 mg/mL) for 2 h at room temperature prior addition to the cells. Each experiment was performed on cells from nine different healthy blood donors.

Raw cells were seeded in 96-well plates at 2 × 10^5^ cells/well in DMEM culture medium (10% heat-inactivated FCS, 50 μg/ml streptomycin, 50 U/ml penicillin) for 24 h. Anti-Apo-A1 IgG (100 μg/ml) were incubated with peptides across a concentration range from 100 μg/ml to 0.06 μg/ml for 2 h at room temperature prior addition to cells. After 24 h stimulation, mouse TNF-α was quantified in the cell supernatants by ELISA according to manufacturer’s instructions (R&D system, MN).

### Statistical analyses

Statistical analyses were performed using *Statistica* software (StatSoft). Association between high immunoreactivity levels and the study endpoint (acute ischemic coronary etiology) is presented as odds ratios (OR) with corresponding 95% confidence intervals. Multivariable analyses using Logistic Regression were used to assess independency between variables. In this model, the endpoint was set as the dependent variable, and Thrombolyis in Myocardial Infarction (TIMI) score for NSTEMI [25] (allowing adjusting for major cardiovascular determinants of 14 days patient outcome within a single continuous variable) was set as the unique confounder due to the limited sample size. Receiver operating characteristic curve (ROC) analyses were performed using *Analyse-It* software for Excel (Microsoft). ROC curve comparison was performed using the Delong method [26]. Unless stated otherwise, results are expressed as median, interquartile range and range. Fisher’s bilateral exact test, Mann—Whitney U-test and Spearman correlation were used when appropriate. P value <0.05 was considered as statistically significant.

## Results

### Exploring the anti-ApoA-I autoantibody response

**Fig 1:**
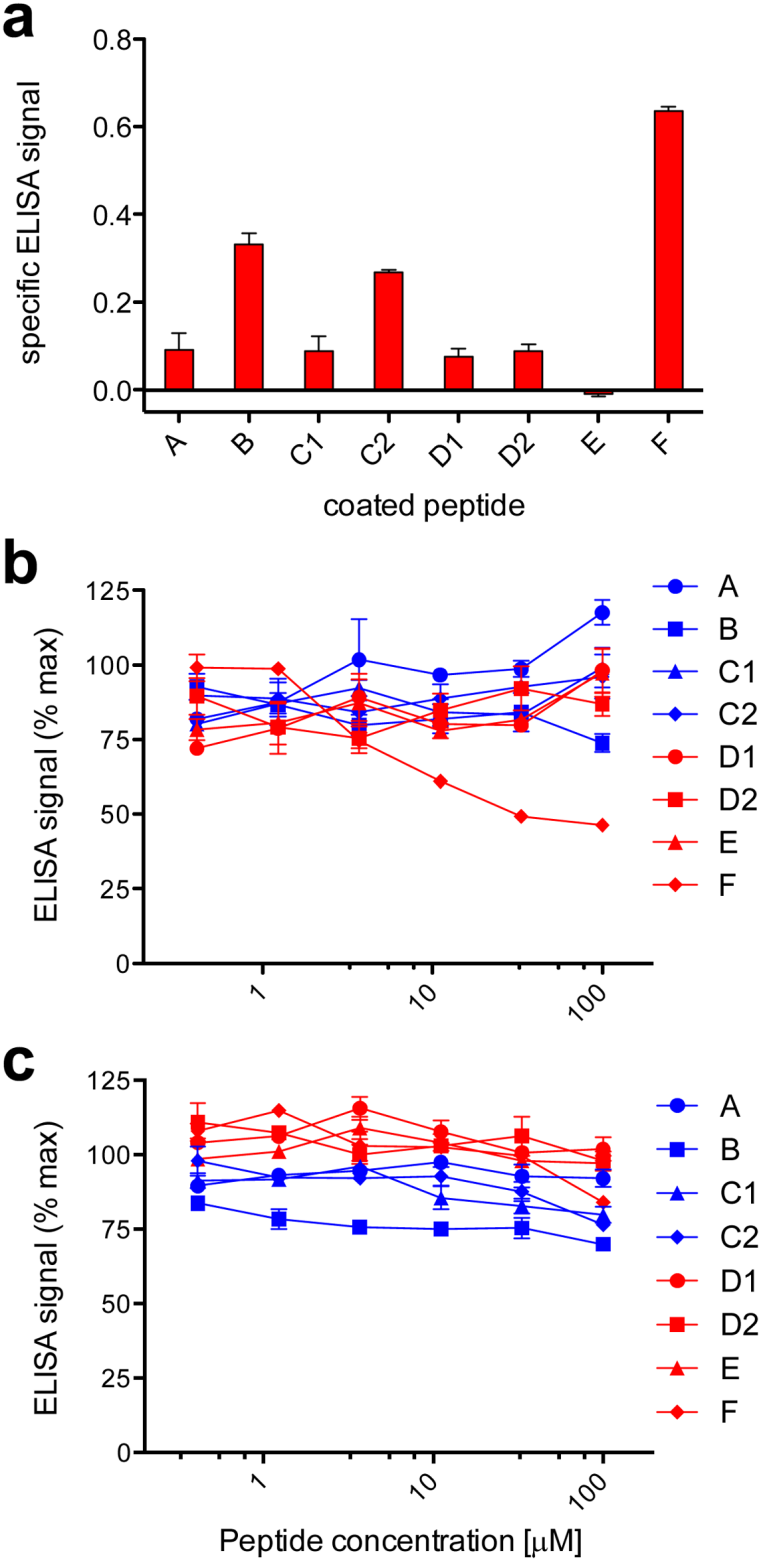
The anti-ApoA-I autoantibody response is strongly biased towards the C-terminal alpha-helical region. ELISA experiments were carried out using a set of ApoA-I-derived peptides (Table 1). a. Capture ELISA assay to determine the immunoreactivity of plasma pooled from patients known to be positive for anti-ApoA-I autoantibodies against the set of peptides. Specific ELISA signals were calculated as [signal in well]-[mean background signal (uncoated well)]. Results are expressed as mean ± SD (n = 3) b and c. Competition ELISA to determine the capacity of the set of peptides to block binding of anti-ApoA-I antibodies from either pooled patient plasma (b) or goat polyclonal IgG (c) to immobilized ApoA-I. Plasma or antibody was preincubated (2 h, room temperature) with peptides at the indicated concentrations prior to addition to assay wells. Percent maximal ELISA signals were calculated as 100 × ([signal in well]-[mean background signal (uncoated well)])/([mean maximal signal (no peptide)]-[mean background signal]). Results are expressed as mean ± SD (n = 3).

In order to characterize the anti-ApoA-I autoantibody response, we chemically synthesized a panel of peptides corresponding to the different alpha-helical regions in the protein (Table 1). Each of the two longest helical regions, C and D, were synthesized as two separate peptides (C1, C2, D1, D2) whose sequences are located on either side of centrally located proline residues (Pro^121^ for Region C, Pro^165^ for Region D (Table 1)). The synthetic peptides were then used as coating antigens in a capture ELISA experiment to compare their immunoreactivity against pooled plasma from a subset of patients from our acute chest pain cohort [14] that had previously shown to be positive for anti-ApoA-I autoantibodies (Fig 1a).

**Table 1:** ApoA-I-derived peptides used in this study. The alpha-helical regions in the lipid-free structure [5] of intact ApoA-I are indicated in italics. The centrally located proline residues in Regions C and D are indicated in bold.

Apo-AI (1–242)		DEPPQSPWD*RVKDLATVYVDVLKDSGRDYVSQFEGSALG*KQLNLKLLDN*WDSVTSTFSKLREQLGPVTQEFWDNLEKETEGLRQ*EMSKDLEEVKAK*VQPYLDDFQKKWQEEMELYRQKVE* *P* *LRAELQEGARQKLHEL*QEKLSPLGE*EMRDRARAHVDALRTHLA* *P* *YSDELRQRLAARLEALKENGGA*RLAEYHAK*ATEHLSTLSEKAKPALED*LRQGLL*PVLESFKVSFLSALEEYTKKLNT*
Peptide	Residues	
A	10–39	RVKDLATVYVDVLKDSGRDYVSQFEGSALG
B	50–84	WDSVTSTFSKLREQLGPVTQEFWDNLEKETEGLRQ
C1	97–120	VQPYLDDFQKKWQEEMELYRQKVE
C2	122–142	LRAELQEGARQKLHELQEKLS
D1	143–164	LGEEMRDRARAHVDALRTHLAPYSDEL
D2	166–187	YSDELRQRLAARLEALKENGGA
E	190–213	ATEHLSTLSEKAKPALED
F	220–242	GLLPVLESFKVSFLSALEEYTKKLNT
F (scrambled)		KELYLLKFTVESKVGSTELPLNFSLA

We noted major differences in plasma immunoreactivity across the panel of peptides, with low signals obtained for Peptides A, C1, D1, D2 and E, intermediate signals for Peptides B and C2, and the strongest signal for Peptide F. Since these differences could have been due to differences in the capacities of the peptides to either (i) adsorb efficiently on assay plates or (ii) present authentic antigenic structures when adsorbed, we performed a second ELISA experiment in which the pooled patient plasma was pre-incubated with the different peptides in solution prior to addition to wells coated with intact ApoA-I (Fig 1b). Since none of the peptides cover more than 15% of the primary sequence of ApoA-I, we would not expect to observe more than 15% inhibition of ELISA signal generated by an unbiased polyclonal antibody response. Notably, Peptide F exhibited detectable dose dependent inhibition in this system, with inhibition at concentrations above 3 μM, reaching a maximal inhibition plateau of approximately 50% of the total anti-ApoA-I signal. In contrast, when pre-incubated with goat polyclonal anti-ApoA-I IgG, none of the peptides, including Peptide F, decreased signal inhibition by 15% at the highest concentration (Fig 1c). Our interpretation of this observation is that while the polyclonal antibody response generated in goats immunized with ApoA-I is unbiased towards any specific region of the protein, the autoantibody response in human subjects is biased towards the C-terminal region corresponding to Peptide F.

### ‘Stapling’ Peptide F to increase alpha-helical content

**Fig 2:**
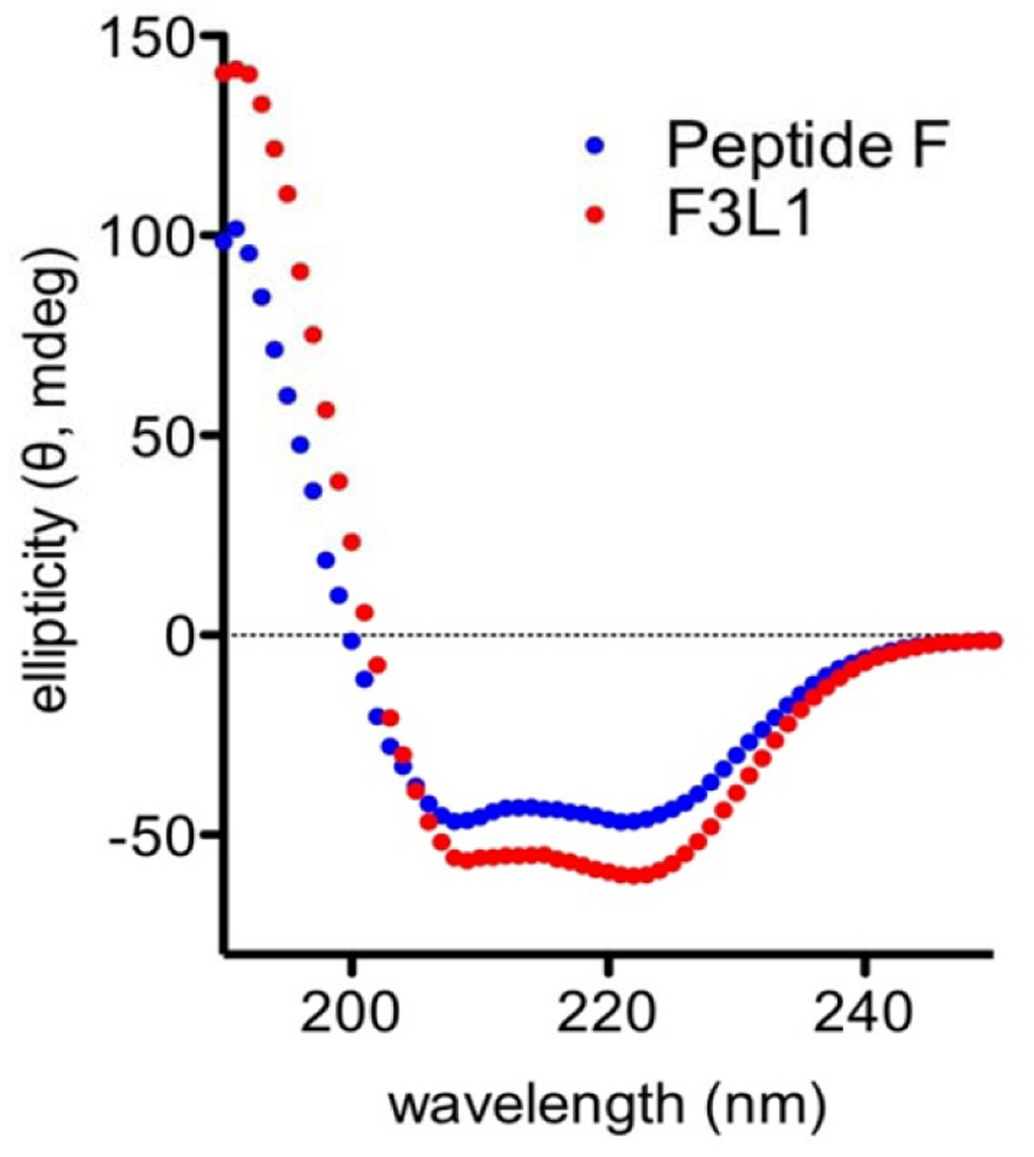
CD spectroscopy of peptide F3L1 indicates increased alpha-helical content. CD spectra of Peptide F and F3L1 were measured at 100μM concentration in water containing 1.25% trifluoroethanol.

We next endeavored to optimize the mimetic qualities of Peptide F by stabilizing its native alpha-helical conformation using a ‘helix-stapling’ approach [24, 27]. We made use of glutamate and lysine residues present three positions apart (i.e. approximately one helix turn) in the sequence of Peptide F to form a lactam bridge. Circular dichroism (CD) spectroscopy analysis showed that the resulting peptide, F3L1 (Ac-GLLPVLESFKVSFLSALEEYTKKLNT-NH_2_; lactam bridge residues underlined), indeed exhibited increased alpha-helical content compared to the parent molecule, Peptide F (Fig 2).

### Anti-ApoA-I immunoreactivity of human plasma samples can be blocked by engineered peptides

**Fig 3:**
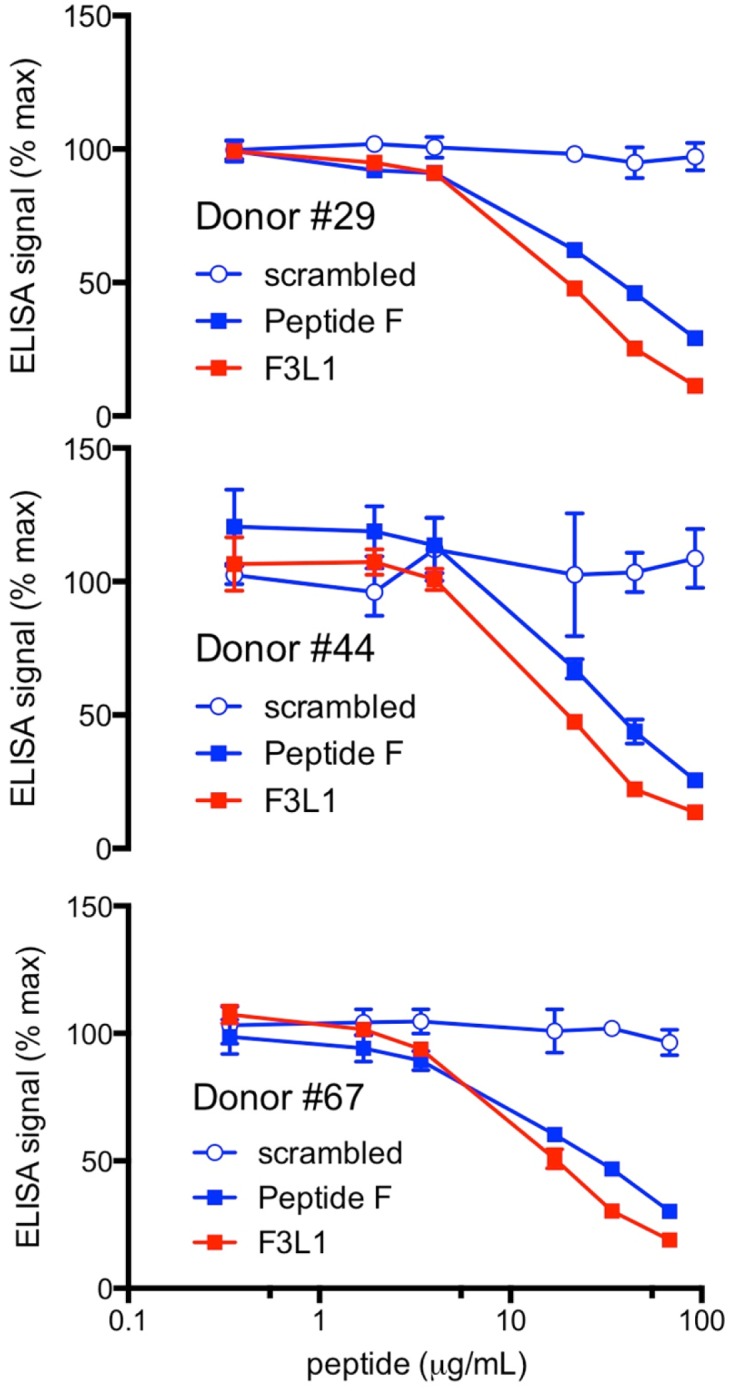
ApoA-I-derived peptides specifically inhibit binding of anti-ApoA-I IgG to immobilized ApoA-I. Competition ELISA to determine the capacity of peptides to block binding of anti-ApoA-I antibodies in plasma samples from three different patients known to be positive for anti-ApoA-I autoantibodies. Plasma samples were preincubated (2 h, room temperature) with peptides at the indicated concentrations prior to addition to assay wells. Percent maximal ELISA signals were calculated as 100 × ([signal in well]-[mean background signal (uncoated well)])/ ([mean maximal signal (no peptide)]-[mean background signal]). Results are expressed as mean ± SD (n = 3).

We next performed ELISA assays using plasma samples from three different anti-ApoA-I autoantibody-positive patients from the cohort pre-incubated with Peptide F, a scrambled version of Peptide F (Table 1), or the stapled peptide F3L1 prior to addition to wells coated with ApoA-I (Fig 3). Peptide F3L1 showed consistently improved binding inhibition with respect to Peptide F. No inhibition was detectable using the scrambled version of Peptide F over the same concentration range.

### Immunoreactivity to Peptide F and F3L1 in human plasma for acute ischemic coronary event diagnosis

To address the clinical relevance of these findings, we next tested whether immunoreactivity to F3L1 in patient serum or plasma could be used for prediction of acute ischemic coronary event diagnosis, as previously demonstrated for NSTEMI using immunoreactivity to full length delipidated ApoA-I [14]. We used our standard in-house immunoassay [14] to assess the potential of Peptide F and F3L1 for use as capture antigens for NSTEMI prediction based on immunoreactivity with plasma samples from a cohort of 132 patients admitted to the emergency room with acute chest pain [14]. The baseline characteristics of these patients are summarized in Table 2.

**Table 2:** 

	Acute Chest Pain Patients (n = 132)	High immunoreactivity to F3L1 (n = 14)	Low immunoreactivity to F3L1 (n = 118)	* *P*
Age in years, median (IQR)	58 (48–70;23–93)	62 (54–68;42–89)	57.5(47–70;23–93)	0.54
**Gender**				
Male, % (n)	63 (83)	71 (10)	62 (73)	0.56
Female, % (n)	37 (49)	29 (4)	38 (45)	-
**Cardiovascular Risk factors**				
Diabetes, % (n)	18 (24)	29 (4)	17 (20)	0.49
Smoking, % (n)	23 (31)	7 (1)	25 (30)	0.26
Dyslipidemia, % (n)	36 (48)	43 (6)	36 (42)	0.63
Obesity, % (n)	14 (19)	7 (1)	15 (18)	0.71
Hypertension, % (n)	43 (58)	57 (8)	42 (50)	0.38
Known CHD, % (n)	28 (37)	36 (5)	27 (32)	0.61
Known Stroke, % (n)	5 (6)	7 (1)	4 (5)	0.86
Positive Familial History, % (n)	11 (15)	7 (1)	12 (14)	0.77
Systolic blood pressure, mmHg	130 (120–150;95–200)	130 (120–154;90–164)	135 (120–148;97–200)	0.69
Diastolic blood pressure, mmHg	75.5 (90–70;50–110)	79.5 (70–80;70–90)	75 (70–90;50–110)	0.66
Heart Rate, bpm	74.5 (65–83;40–170)	72.5 (64–78.5;47–97)	74.5 (65.5–84;40–170)	0.56
Body Mass Index, kg/m^2^	26.1 (23.9–29.4;16.4–38.3)	26.7 (23.6–29.4;21.9–32.3)	26.0 (23.9–29.4;16.4–38.3)	1
NSTEMI-TIMI score at admission	2 (1–3;1–6)	3 (2–4;1–5)	2 (1–3;1–6)	0.03
**Medical Treatment Upon Admission**				
Aspirin, % (n)	39 (52)	71 (10)	36 (42)	0.01
Clopidogrel, % (n)	8 (11)	7 (1)	8 (10)	0.95
β-blockers, % (n)	28 (37)	43 (6)	26 (31)	0.27
ACE inhibitors, % (n)	24 (32)	36 (5)	23 (27)	0.38
AT-1 blockers, % (n)	9 (12)	14 (2)	8 (10)	0.7
Insulin, % (n)	5 (7)	0 (0)	6 (7)	0.72
Oral anti-diabetic agents, % (n)	20 (26)	29 (4)	17 (22)	0.5
Diuretics, % (n)	17 (23)	29 (4)	16 (19)	0.41
Calcium Channel Blockers, % (n)	11 (14)	7 (1)	11 (13)	0.82
Statins, % (n)	32 (42)	50 (7)	30 (35)	0.1
**Biological Parameters Upon Admission**				
Total Cholesterol, mmol/l	4.4 (3.9–5;2.7–6.7)	4.2 (3.5–4.4;3–5.2)	4.5 (4–5; 2.7–6.7)	0.33
HDL, mmol/l	1.05 (0.79–1.28;0.65–2.29)	0.88 (0.77–1.53;0.7–1.65)	1.09 (0.91–1.28;0.65–2.29)	0.65
LDL, mmol/l	2.58 (2.1–3.15;0.71–4.56)	2.81 (1.59–3.03;1.21–14.6)	2.57 (2.10–3.15;0.71–4.56)	0.79
Triglycerides, mmol/l	1.41 (0.89–2.14;0.3–5.1)	1.31 (0.84–1.53; 0.3–2.38)	1.48 (0.96–2.14;0.44–5.09)	0.4
Creatinine, μmol/L	78.5 (67–89;45–131)	97 (62–127;51–196)	77.5 (65.5–88.5;39–285)	0.92
GFR, mL/min	70 (60–110;14–202)	60 (52–91.5;29–167)	72 (60–111;14–202)	0.28
CRP, mg/L	3 (1–10; 0–274)	5.5 (3–10;1–23)	3 (1–10;0–274)	0.21
Initial cTnI value, ng/ml	0.02 (0.01–0.04; 0–23)	0.05 (0.01–0.13;0.005–3.5)	0.02 (0.01–0.036;0–23)	0.08
**Diagnostic at Discharge, % (n)**				
*Acute Ischemic Coronary event (NSTEMI/UA)*	22 (29)	50 (7)	19 (22)	0.002
*Non Ischemic Etiology*:	78 (103)	50 (7)	93 (110)	-
Parietal etiology	7 (9)	7 (1)	-8	-
Gastroenterological etiology	5 (7)	14 (2)	-5	-
Pulmonary etiology	2 (3)	0	-3	-
Supraventricular Arrhythmia	3 (4)	0 (0)	-4	-
Pulmonary embolism	2 (3)	7 (1)	-2	-
Pericarditis	2 (2)	0 (0)	-2	-
Psychogenic	2 (3)	0 (0)	-3	-
Malignant hypertension	1 /1)	0 (0)	-1	-
Unknown, but pulmonary embolism and aortic dissection ruled-out	59 (78)	21 (3)	60 (75)	-

*P value was computed by comparing patients with high vs low anti-F3L1 immunoreactivity. CHD: coronary heart disease, bpm: beats per minute, GFR: glomerular filtration rate.

**Fig 4:**
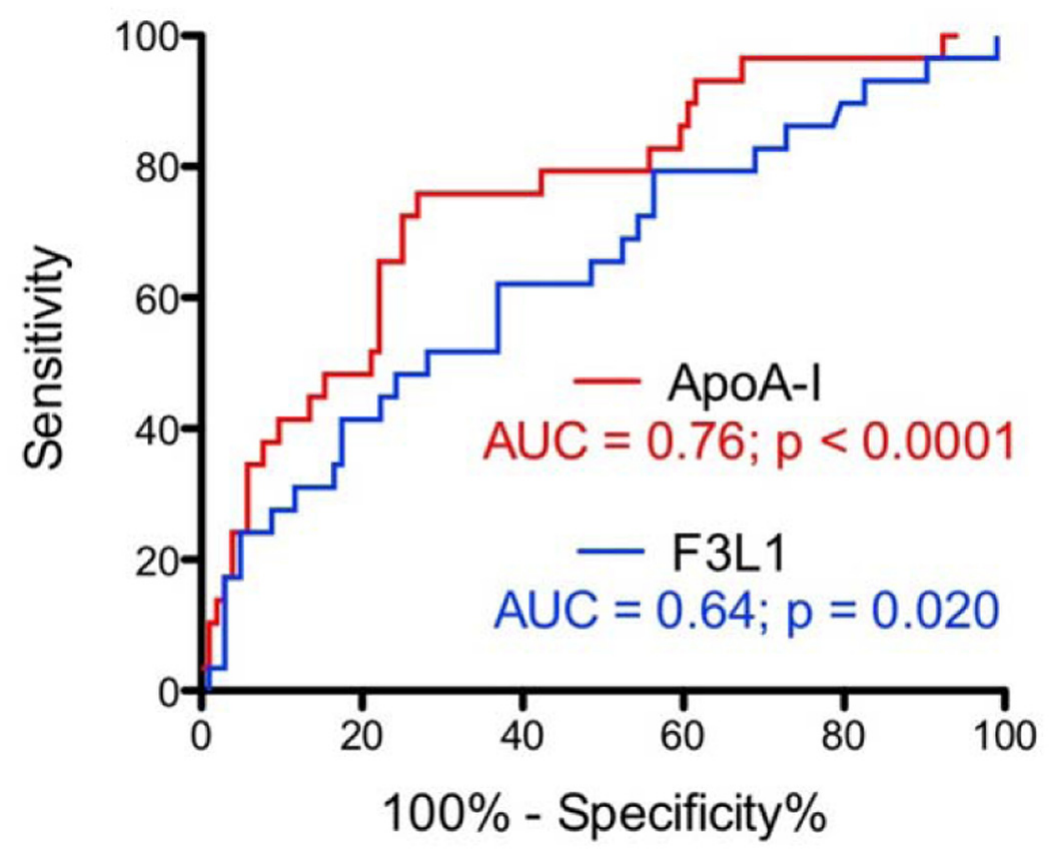
Predicitve accuracy for acute ischemic coronary events of immunoreactivity to F3L1 and to native ApoA-I.

Patients with high immunoreactivity to F3L1 were found to be more likely under aspirin treatment, to have a higher NSTEMI-TIMI score, and to have an acute ischemic coronary etiology upon discharge when compared to patients with low immunoreactivity to F3L1 (Table 2). As expected from the previous study [14], using intact ApoA-I as a coating antigen provided statistically significant prediction for an acute ischemic coronary event (area under curve (AUC) 0.75; 95% confidence interval (CI) 0.64–0.85; p <0.0001). While Peptide F did not provide statistically significant prediction of NSTEMI (AUC 0.55; 95% CI 0.41–0.68; p = 0.25), peptide F3L1 provided results approaching those obtainable with the intact protein (AUC 0.64; 95% CI 0.52–0.76; p <0.01) (Fig 4), although the AUC difference between endogenous apoA-I and F3L1 was found to be statistically significant (p = 0.03).

Translated into risk analyses, positive patient plasma immunoreactivity to F3L1 was associated with a 4-fold risk of an acute ischemic coronary event (univariate OR: 4.36, 95% CI: 1.38–13.71, p = 0.01), independently of the adjustment for the NSTEMI-TIMI score (OR: 3.61; 95%CI: 1.01–13.00, p = 0.04). In comparison, positive immunoreactivity to native ApoA-I increased the risk of an acute ischemic coronary event by 11-fold (OR 10.88; 95%CI: 2.64–26.76, p = 0.004) and by 7-fold (OR: 6.94, 95%CI: 3.32–35.62, p<0.001) after the adjustment for the NSTEMI-TIMI score. Immunoreactivity to Peptide F was not statistically significantly associated with an increased risk of acute ischemic coronary event (data not shown).

Finally, Spearman correlation showed positive and significant associations between antibodies to F3L1 and CRP (r = 0.23, p = 0.03), and the first cTnI value available upon patients’ admission (r = 0.18, p = 0.04).

### ApoA-I-derived peptides block anti-ApoA-I IgG-mediated proinflammatory cytokine release

**Fig 5:**
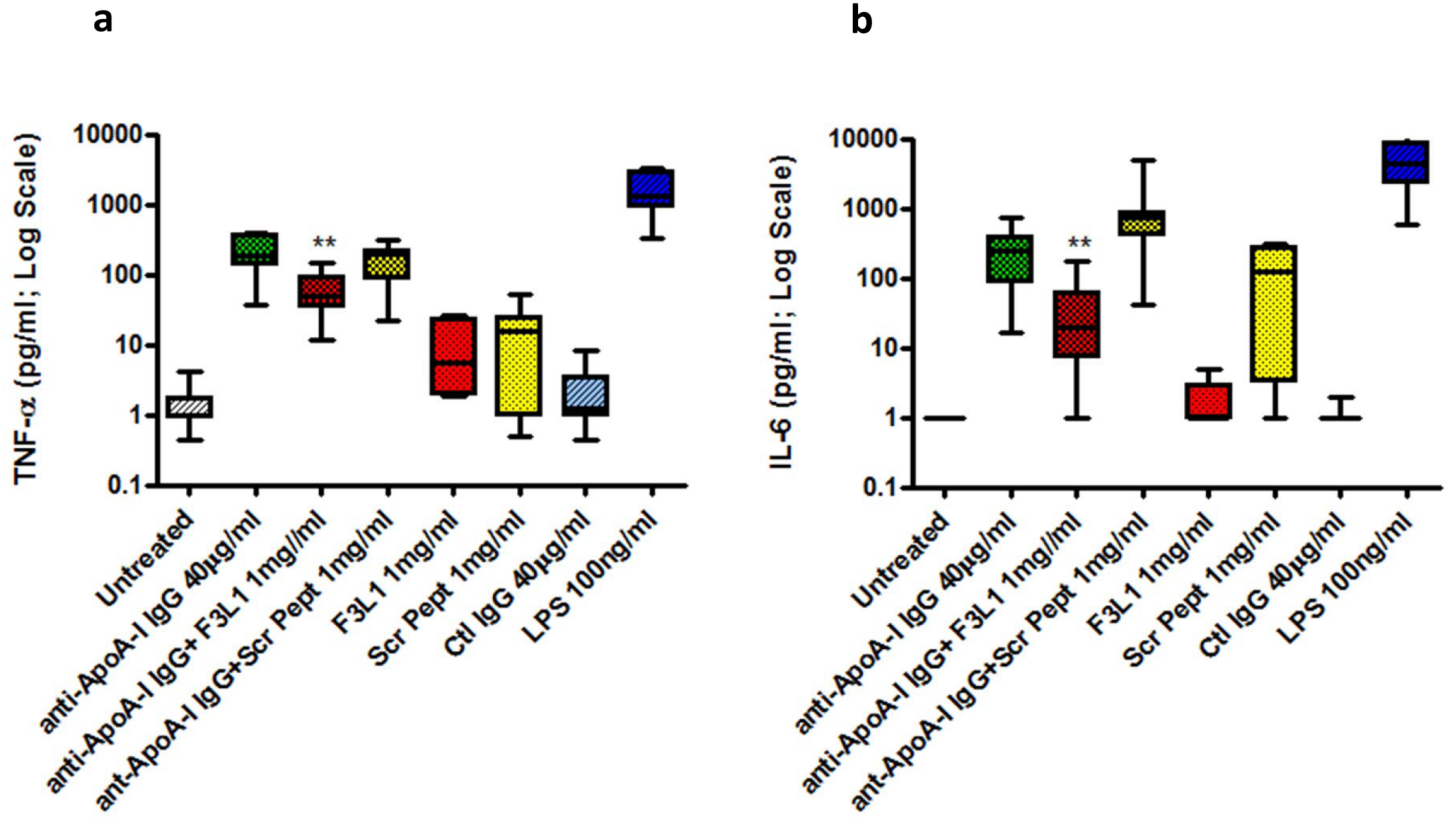
Peptide F3L1 inhibits anti-ApoA-I IgG-mediated release of proinflammatory cytokines from cultured human monocyte-derived macrophages. Release of proinflammatory cytokines TNF-α (**a**) IL-6 (**b**) from human monocyte-derived macrophages induced by endotoxin-free goat polyclonal anti-ApoA-I IgG was determined with or without preincubation (2 h at room temperature) of the anti-ApoA-I IgG with peptide F3L1 (1 mg/mL) and the corresponding scrambled peptide (Scr Pept). Experiments were repeated using cells from nine different healthy donors, with results expressed as median, interquartile range (IQR) and range. ** p value <0.008.

Both plasma from human myocardial infarction patients known to be positive for anti-ApoA-I autoantibodies and anti-ApoA-IgG from immunized animals have been shown to elicit a dose-dependent release of proinflammatory cytokines from human primary macrophages with an optimal response at 40 μg/ml [12, 15, 19]. We next used a Luminex assay to measure the capacity of peptide F3L1 to inhibit endotoxin-free anti-ApoA-I IgG-mediated proinflammatory cytokine release in human monocyte-derived macrophages (Fig 5a and 5b). As expected from previously published work [12, 15, 19], anti-ApoA-I IgG elicited a significant increase in production of both in IL-6 and TNF-α when compared to control IgG (Ctl IgG; p<0.0001 for both cytokines). Release of these cytokines was strongly inhibited when the anti-ApoA-I IgG (40μg/ml) was pre-incubated with peptide F3L1 (1 mg/mL, 342 μM) prior to addition to cells, decreasing the median IL-6 production from 250.9 to 19.2 pg/ml (p = 0.008), and the median TNF-α production from 183.7 to 48.4 pg/ml (p = 0.028). On the other hand, co-incubating anti-ApoA-I IgG with the corresponding scrambled peptide did not inhibit the pro-inflammatory response induced by the anti-ApoA-I antibodies. When compared to control IgG, F3L1 was devoid of significant biological activity in terms of cytokine production, although a trend was observed for TNF-α production (p = 0.05; Fig 5a and 5b). We were unable to measure the inhibitory activity of intact purified and delipidated ApoA-I in this assay because traces of endotoxin elicit significant levels of background cytokine secretion (NV and SP, unpublished observations).

**Fig 6:**
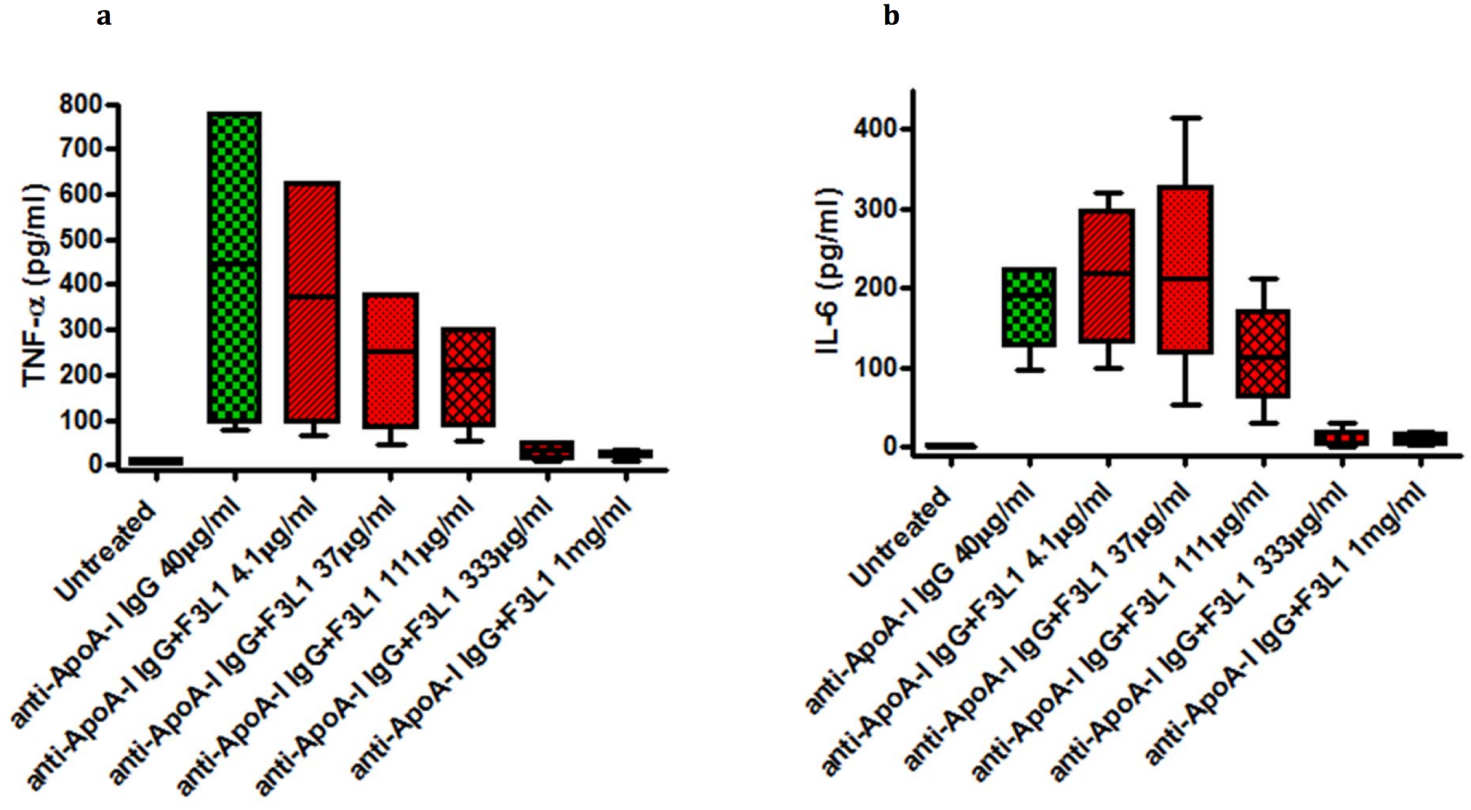
Peptide F3L1 dose-dependently inhibits anti-ApoA-I IgG-induced TNF-α and IL-6 production. Anti-ApoA-I IgG was incubated with the indicated F3L1 concentrations (preincubation 2 h at room temperature) prior to addition to cultured human monocyte-derived macrophages. Levels of proinflammatory cytokines were determined after 24 h culture. Experiments were repeated using cells from three different healthy donors, with results expressed as median, interquartile range (IQR) and range. Kruskal-Wallis test for a trend showed p value = 0.01 for TNF-α, and p value = 0.005 for IL-6.

**Fig 7:**
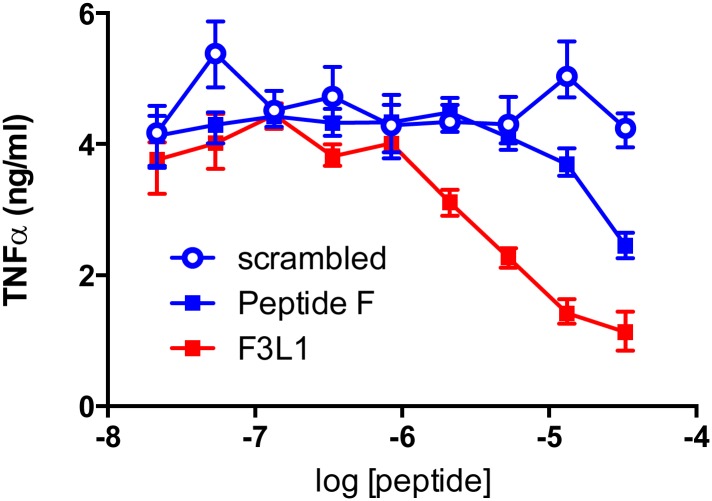
Comparing the inhibitory potency of Peptide F and F3L1 on anti-ApoA-I IgG-induced TNF-α. Anti-ApoA-I IgG was incubated with the indicated peptide concentrations (preincubation 2 h at room temperature) prior to addition to cultured RAW cells. Levels of proinflammatory cytokines (TNF-α) were determined after 24 h culture. Mean levels (n = 3) are shown with error bars indicating the range. Kruskal-Wallis test for a trend showed p value = 0.01.

As shown in Fig 6a and 6b, anti-ApoA-I IgG-induced TNF-α and IL-6 release in human monocyte-derived macrophages was inhibited by F3L1 in a dose-dependent manner. When used at the highest concentration (1 mg/mL) in this model, Peptide F provides a level of inhibition of anti-ApoA-I IgG-mediated cytokine release comparable to that of F3L1. To compare the potency of the stapled versus the unstapled peptide, we performed dose-response experiments for inhibition of anti-ApoA-I IgG-mediated cytokine release in the RAW mouse macrophage cell line (Fig 7). In this system, F3L1 is clearly a more potent inhibitor than Peptide F, with the scrambled peptide showing no detectable inhibitory activity.

## Discussion

### Strong bias in the human anti-ApoA-I IgG autoantibody response towards the C-terminal alpha-helix

Our results indicate that the anti-ApoA-I IgG autoantibody response is strongly biased towards the C-terminal alpha-helix (Fig 1a and 1b). These findings, obtained with pooled patient plasma, were recapitulated in plasma samples obtained from three different individual patients, where an optimized ApoA-I mimetic peptide corresponding to amino acids 240–267 (i.e. covering less than 11% of the total linear sequence of the protein) was capable of neutralizing up to 90% of the total anti-ApoA-I IgG signal (Fig 3). Our observations confirm and extend findings obtained in a previous study in which peptide fragments were obtained by proteolytic digestion of purified endogenous ApoA-I [28]. While in this previous study immunoreactivity was noted to a long peptide fragment corresponding to residues 141–182 of ApoA-I, we did not detect binding to two shorter peptides that partially cover this region, Peptide D1 (143–164) and Peptide D2 (166–187). The simplest explanation for this finding is the anti-ApoA-I autoantibodies that bind to the longer peptide recognize an epitope that is not entirely present in either of the two shorter peptides. Together, these studies provide compelling support for the idea that the anti-ApoA-I IgG autoantibody response is strongly focused towards an epitope on ApoA-I corresponding to the C-terminal alpha-helix, but in order to robustly test this hypothesis it would be necessary to screen a larger number of individual patient samples.

### Characteristics of the C-terminal alpha-helix epitope of ApoA-I

Although the peptides used in this study were designed based on the structure of lipid-free ApoA-I, it is likely that the epitope or epitopes present on Peptide F that are engaged by anti-ApoA-I antibodies are also present and available for interaction on lipid-associated ApoA-I. According to structural studies of lipid-associated and lipid-free ApoA-I (reviewed in [6]), the region corresponding to Peptide F is always present in an alpha-helical conformation, with its hydrophilic face consistently oriented towards the aqueous exterior, and its hydrophobic face either packed on to the hydrophobic face of another helical region of the protein (in the lipid-free form) or on to the lipid surface (in both HDL forms). Importantly, since peptides corresponding to this region do not show enhanced neutralization of the anti-ApoA-I signal of polyclonal IgG from an immunized animal (Fig 1c), it is unlikely that the C-terminal alpha-helix of ApoA-I is favored in the autoantibody responses in humans because it contains intrinsically immunodominant epitopes. While the mechanisms by which anti-ApoA-I antibodies arise remain elusive, the focused nature of the anti-ApoA-I autoantibody response (this study and [28]) could be an indication for the involvement of pathogen molecular mimicry i.e. generation of antibodies against a pathogen molecule that is structurally similar to the region of ApoA-I corresponding to Peptide F. Pathogen molecular mimicry has been put forward as an explanation for the generation of autoantibodies related to other pathologies [29–31], and could explain how anti-ApoA-I IgG can arise in subjects who do not show any signs of genuine loss of self-tolerance [14–17].

### Use of ApoA-I-mimetic peptides based on the C-terminal alpha-helix epitope as tools for prognosis and diagnosis of atherosclerosis and CVD

Detecting circulating levels of anti-ApoA-I autoantibodies in patients is a promising strategy for developing new assays for risk stratification in atherosclerosis and CVD [12, 14–18, 32], and the evidence for bias in the anti-ApoA-I autoantibody response towards the C-terminal helix of the protein provides a rationale for developing peptide-based prognostic and diagnostic tests based on this region. Indeed using an optimized mimetic peptide to diagnose the occurrence of an acute ischemic coronary event (non-ST segment elevation myocardial infarction and unstable angina) on a cohort of acute chest pain patients, we were able to approach the diagnostic accuracy obtainable using intact endogenous ApoA-I. Further optimization of the peptide antigen, together with optimization of its immobilization in the immunoassay, should enable improvements in diagnostic accuracy from this starting point.

### Use of ApoA-I-mimetic peptides based on the C-terminal alpha-helix epitope to block the pathogenic activity of anti-ApoA-I IgG

There is increasing evidence to suggest that anti-ApoA-I autoantibodies play a direct role in the pathogenesis of atherosclerosis and CVD and as such should be considered as targets for therapeutic intervention [12, 14–18]. Here we have shown that peptides from the C-terminal region of ApoA-I strongly inhibit one of the pathogenesis-associated activities of anti-ApoA-I autoantibodies, release of proinflammatory cytokines from cultured macrophages (Figs 5, 6 and 7), with F3L1, the stapled version of Peptide F, showing clearly improved inhibitory potency (Fig 7). Hence we have shown that optimized ApoA-I-derived peptides can inhibit the anti-ApoA-I IgG-mediated inflammatory response in vitro, but whether this will translate into blockade of pro-atherogenic effects *in vivo* [15] remains to be demonstrated.

Notably, the almost complete inhibition achieved by F3L1 of goat anti-ApoA-I IgG-mediated pathogenesis-associated activity suggests that antibodies directed towards the C-terminal alpha-helical region of ApoA-I are primarily responsible for the previously described proinflammatory effects mediated by anti-ApoA-I IgG [12, 14–18]. This observation is of relevance to the elucidation of the structural specificity of pathogenesis-associated anti-ApoA-I antibodies [19].

Given the fact that F3L1 does not contain the LCAT activation site (residues 165–206 of ApoA-I) [8] and that immunoreactivity to peptides covering this regions were devoid of significant diagnostic accuracy, it is likely that antibodies to F3L1 do not interfere with cholesterol efflux. These results are in line with unpublished results from our group showing that sera from patients containing high levels of anti-apoA-I IgG do not interfere with cholesterol efflux in vitro.

Finally, with respect to the disputed clinical efficacy of ApoA-I mimetic peptides administered to unselected high-risk patients [33–35], our observations support the hypothesis that restricting the administration of such compounds to patients with high levels of anti-apoA-I IgG could improve the efficiency and the individualized nature of ApoA-I mimetic peptide-based therapy. Nevertheless, given the fact that the extrapolation of *in vitro* data to human physiopathology in the field of HDL and ApoA-I has been known to be particularly difficult [8], this appealing hypothesis requires to be demonstrated.

### Perspectives for further optimization of ApoA-I-mimetic peptides

We have shown that the capacity of ApoA-I-mimetic peptides to bind to (Fig 3) and neutralize (Fig 7) the effects of anti-ApoA-I autoantibodies can be improved through the use of a ‘helix staple’ to enhance alpha-helical content, and it is likely that further optimization will be possible from this starting point. Firstly because some of the epitopes recognized by the anti-ApoA-I autoantibodies may have been masked by the structure of the lactam staple used in F3L1, and secondly because further constraint into the native alpha-helical conformation is likely possible. Indeed, several other helix-stapling approaches have been described [24, 27, 36] in addition to lactam bridge formation; changing the nature, position and number of helix staples should enable identification of the optimal combination to minimize epitope masking and maximize conformational constraint.

### Scope for future work to address the limitations of this study

One limitation of this study is the small study sample size of our acute chest pain cohort. In future work it will be important: (i) to replicate and validate these preliminary results at larger multicenter scale, and (ii) to determine whether the diagnostic accuracy of immunoreactivity to F3L1 or other optimized peptides is equivalent to the one obtained with endogenous ApoA-I, and whether it could provide incremental diagnostic information over high-sensitive troponin assays. A second important limitation is that STEMI patients were excluded from this study. The rationale for excluding these patients relates to the fact that biomarkers in general are only of marginal value in the early management of STEMI, whereas they represent the cornerstone of MI diagnosis in NSTEMI settings [22]. Since NSTEMI patients are known to be older, to have a higher number of co-morbidities, and to be at greater risk of developing CV complications after acute MI than STEMI patients [37], further large-scale studies that include both STEMI and NSTEMI patients will be required in order to completely define the prognostic value of those autoantibodies for cardiovascular disease. Such studies would also allow the value of using levels F3L1-reactive antibodies for risk stratification in primary prevention settings to be assessed.

### Conclusion

In this study we have provided evidence that the anti-ApoA-I autoantibody response in humans is biased towards the C-terminal alpha-helical region of the protein. In addition to providing insights into the mechanism by which anti-ApoA-I autoantibodies are elicited in subjects without autoimmune disease, this observation provides a rationale for the development of new ApoA-I mimetic peptides with potential use in both diagnosis and targeted therapy of atherosclerosis and CVD.
